# A Study of Factors Leading to Difficult Laparoscopic Cholecystectomy at a Tertiary Care Center in Northeastern India

**DOI:** 10.7759/cureus.74218

**Published:** 2024-11-22

**Authors:** Noor Topno, Donkupar Khongwar, Girish Sharma, Baphiralyne Wankhar, Arup Baruah, Dathiadiam Tongper, Sandeep Ghosh, Narang Naku, Yookarin Khonglah, Ranendra Hajong, Polina Boruah

**Affiliations:** 1 General Surgery, North Eastern Indira Gandhi Regional Institute of Health and Medical Sciences, Shillong, IND; 2 Urology, Max Super Speciality Hospital, Delhi, IND; 3 Radiology and Medical Imaging, University of Virginia, Charlottesville, USA; 4 Surgical Oncology, North Eastern Indira Gandhi Regional Institute of Health and Medical Sciences, Shillong, IND; 5 General Surgery, Tomo Riba Institute of Health and Medical Sciences, Naharlagun, IND; 6 Pathology, North Eastern Indira Gandhi Regional Institute of Health and Medical Sciences, Shillong, IND; 7 Biochemistry, North Eastern Indira Gandhi Regional Institute of Health and Medical Sciences, Shillong, IND

**Keywords:** difficult cholecystectomy, intraoperative factors, laparoscopic cholecystectomy, non difficult cholecystectomy, north eastern indira gandhi regional institute of health and medical sciences, open cholecystectomy, patient factors, ultrasonographic factors

## Abstract

Background: Laparoscopic cholecystectomy (LC) is currently the gold standard of care for managing gallstone disease. The time taken to perform LC depends on both patient-related and surgeon-related factors. Recognizing factors associated with difficult LC (DLC) can aid in appropriate surgeon selection and judicious scheduling of cases.

Methods: This prospective study was conducted to identify preoperative factors (clinical and ultrasonographic) and intraoperative factors that can help predict or prepare for DLC. The study took place in the Department of General Surgery, North Eastern Indira Gandhi Regional Institute of Health and Medical Sciences, Shillong, India. A total of 100 cases of LC were enrolled over a two-year period. All patients had symptomatic cholelithiasis and were scheduled to undergo elective LC. The time taken to perform LC was compared against individual parameters of interest, including clinical features, ultrasonography (USG), and intraoperative findings.

Results: Forty-one LCs were classified as difficult and 59 as non-DLC (NDLC), based on the time limit set by the mean operating time for all LC cases. Seven out of the 41 difficult LCs required conversion to open cholecystectomy (OC). Patient, USG, and intraoperative factors were found to have a significant correlation with difficult LC. Patient factors included male gender, body mass index (BMI), number of past attacks, and previous abdominal surgery. USG factors included calculi number, calculi size, impaction of calculi, and a thick gallbladder (GB) wall. Intraoperative factors included pericholecystic adhesions, Calot’s triangle dissection, GB mobilization from the liver bed, and GB specimen extraction.

Conclusion: Preoperative identification of difficult LC cases can guide rational allocation of cases based on surgeon experience, leading to better utilization of operating theatre time and reducing the probability of conversion and complications.

## Introduction

Laparoscopic cholecystectomy (LC) is one of the highest-volume surgeries performed annually [1]. Difficult LC (DLC) is a challenging condition, and its definition is not well-established, often varying according to the surgeon's experience. Although LC is now considered the optimal surgical procedure for symptomatic gallbladder (GB) lithiasis, it should not be underestimated, as vascular and biliary duct injuries are highly morbid, significantly increase healthcare costs, and often lead to litigation [2].

Surgeons may encounter difficulty during LC when there are dense adhesions in Calot’s triangle, a fibrotic and contracted GB, an acutely inflamed or gangrenous GB, bilio-enteric fistula, among other conditions. Several risk factors can make LC difficult, including advanced age, male sex, episodes of acute cholecystitis with fever and leukocytosis, obesity, prior abdominal surgery, and specific ultrasonographic findings such as a thickened GB wall, distended GB (mucocele), pericholecystic fluid collection, and impacted stones [3,4].

The frequency of complications associated with laparoscopic cholecystectomy ranges from 0.5% to 6% [5,6]. Conversion to open cholecystectomy (OC) may be required for various reasons, with reported rates of 1% to 15% [7,8].

While LC is generally safe and effective, it can occasionally present challenges. Issues may include difficulty in creating pneumoperitoneum, accessing the peritoneal cavity, releasing adhesions, identifying anatomical structures and anatomical variations, and extracting the GB. LC procedures that encounter such issues and take longer than usual are classified as difficult [9].

Studies examining factors associated with difficult LC have yielded conflicting results. Our research aimed to identify factors that can help predict, anticipate, and prepare for difficult LCs, focusing on the spectrum of patients in our region. Additionally, we sought to familiarize junior surgeons with the preoperative and intraoperative warning signs of a probable DLC and to encourage the adoption of bailout strategies when confronted with a difficult GB, always adhering to the principle of "first, do no harm."

## Materials and methods

A prospective non-randomized analytical cross-sectional study was conducted on 100 consenting adult patients with symptomatic cholelithiasis undergoing elective LC from 2019 to 2021 in the Department of General Surgery at North Eastern Indira Gandhi Regional Institute of Health and Medical Sciences (NEIGRIHMS), Shillong. The operating surgeon was blinded to preoperative patient details (clinical and USG). The Institution Ethics Committee approved the study.

Inclusion criteria were as follows: (1) adult patients aged 18 years or older with symptomatic cholelithiasis scheduled for interval cholecystectomy and (2) patients undergoing standard four-port LC.

Exclusion criteria were as follows: (1) patients unwilling to provide consent for participation in the study, (2) patients with concurrent common bile duct stones, (3) patients with acute cholecystitis, (4) LC performed with other interventions, and (5) cholelithiasis in pregnancy.

Patients presenting with acute cholecystitis were treated conservatively. Conservative management included nil per oral, intravenous hydration, complete hemogram, and pain relief. Antibiotics were administered on a case-by-case basis based on clinical features and laboratory indices for infection. In addition to USG, cross-sectional imaging was performed when indicated (e.g., serum pancreatic enzyme levels). Patients were discharged after symptom resolution and normalization of laboratory parameters (e.g., total leukocyte count), typically within 48 to 72 hours. Readmission for elective LC was scheduled six weeks after hospital discharge. LC was performed within one week following endoscopic retrograde cholangiopancreatography (ERCP) to reduce the risk of recurrent biliary events.

For all study participants, relevant history was elicited, and a clinical examination was performed. Ultrasonography was conducted by a designated consultant radiologist one day before the operation, with findings recorded in a pre-designed proforma. Informed consent for the operation (LC +/- conversion to OC) and pre-anesthetic clearance were obtained for all patients. All patients received general anesthesia and a prophylactic dose of cefuroxime 1.5 g at induction. Admission, treatment, consent, and demographic details were recorded in a pre-designed proforma and maintained by the author.

Each LC was performed by experienced consultant surgeons who had completed at least 200 LCs independently and had more than 10 years of experience in laparoscopic surgery. LC was performed using the classical four-port technique with the patient in a supine position, reverse Trendelenburg, and right-side-up lateral tilt configuration. Pneumoperitoneum was achieved either by Veress needle or, in a few cases, by the Hasson open technique, with a pneumoperitoneum pressure range of 10-12 mm Hg. Dissection was performed using Maryland forceps or a monopolar energy device. The cystic duct and cystic artery were secured with metal clips. The same laparoscopic system and hand instruments were used for all cases. No additional ports were used. The high-definition laparoscopic system was supplied by Karl Storz SE & Co. KG (Tuttlingen, Germany).

Conversion to OC or performing a subtotal cholecystectomy was at the discretion of the operating surgeon. The GB was extracted via the epigastric port using a readymade specimen retrieval device (WEL-EndoBag®, Well Lead Medical, Guangzhou, China). Tube drain placement was based on the surgeon’s decision. Closure of 10 mm ports included fascial layer closure with 1/0 Polyglactin 910 and skin closure with 3/0 nylon sutures, while only skin was closed for 5 mm ports. LC procedure time was recorded for all patients from the time of Veress needle insertion/skin incision (for Hasson open access) to the time of complete GB retrieval. Postoperatively, the surgeon recorded any difficult steps or conditions encountered during the LC.

Data analysis

Each parameter of the preoperative clinical assessment, ultrasonography findings, and intraoperative findings was recorded and analyzed for a correlation with operating time. The mean operating time calculated for all LCs (n = 100) was 61.46 minutes. LCs taking more time than this duration were categorized as difficult laparoscopic cholecystectomy (DLC). The remaining LCs completed within 61.46 minutes were considered non-DLC (NDLC). For converted cases, the operating time recorded was from the moment of Veress needle insertion to the time of the decision to convert to OC. The duration of the OC was not counted.

The chi-square test was used to differentiate between statistically significant and non-significant associations between variables of interest and LC. The p-value for NDLC versus DLC against a particular variable was calculated, with a p-value of less than 0.05 considered indicative of a significant association. IBM SPSS Statistics for Windows, Version 21 (Released 2012; IBM Corp., Armonk, New York).

## Results

**Figure 1:**
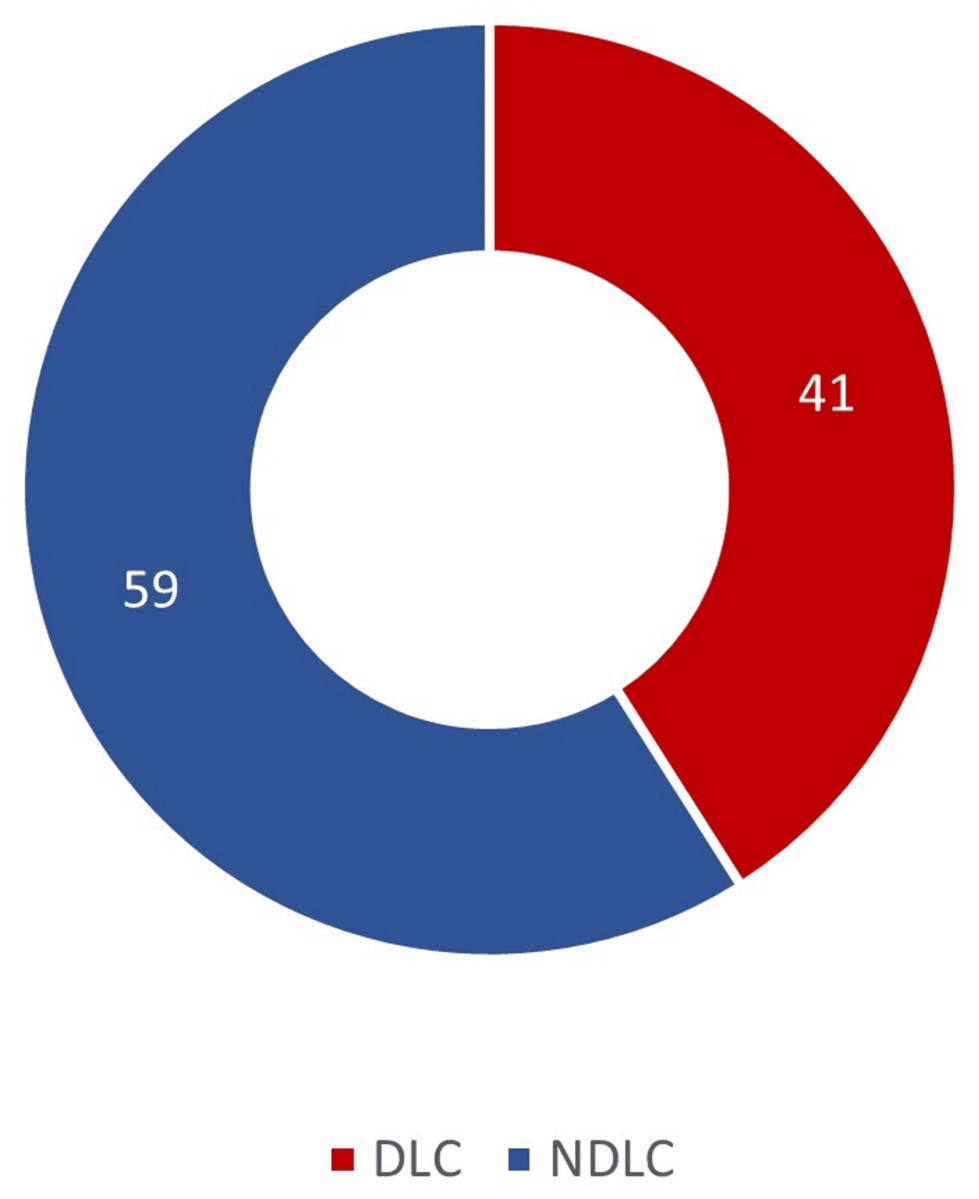
Distribution of DLC and NDLC cases. DLC: difficult laparoscopic cholecystectomy, NDLC: non-difficult laparoscopic cholecystectomy.

One hundred patients were enrolled, with 41 LCs taking more time than the calculated mean operating time of 61.46 minutes. These cases were considered difficult LC, with a mean operating time of 86 minutes, and were referred to as the "DLC group." The operating time for 59 patients was less than the calculated mean of 61.46 minutes; these were considered NDLC, with a mean operating time of 44.37 minutes (Figure 1). This set of patients was termed the "NDLC group." Since the total number of cases was 100, the numerical value of/against a particular variable is equivalent to a percentage. Seven cases were converted to OC due to intraoperative difficulty. No complications, reoperations, or mortality occurred in any of the patients.

Most patients were in the age group of 31-40 years. The youngest and oldest patients were 19 and 65 years, respectively. The study population comprised 91 female and 9 male patients.

Nine male patients were present in the study population. Seven patients had DLC, while two had NDLC. A significant association between male gender and DLC was noted, with a p-value of 0.0459 (Table 1).

**Table 1:** Clinical parameters assessed for difficult LC. DLC: difficult laparoscopic cholecystectomy, NDLC: non-difficult laparoscopic cholecystectomy, BMI: body mass index, LC: laparoscopic cholecystectomy, ERCP: endoscopic retrograde cholangiopancreatography.

Clinical factor	Number of patients	DLC	NDLC	p-value
Male gender	9	7	2	0.0459
BMI (>30 kg/m^2^)	17	12	5	0.0130
<6 weeks interval between LC and attack of cholecystitis	47	23	24	0.1882
≥2 attacks of cholecystitis	54	33	21	0.0001
Diabetes mellitus	4	1	3	0.8845
History of ERCP	4	3	1	0.1761
History of biliary pancreatitis	3	2	1	0.7476
History of abdominal surgery	33	19	14	0.0316

A BMI greater than 30 kg/m² was noted in 12 patients with DLC and five patients with NDLC. The remaining patients in the DLC group had a BMI of less than 30 kg/m². A p-value of 0.0130 was calculated, suggesting a statistically significant association between BMI > 30 kg/m² and DLC (Table 1).

Cholecystitis at least six weeks before the operation was observed in 47 patients: 23 patients had DLC and 24 patients had NDLC. Among the 53 patients who did not have cholecystitis within six weeks prior to the operation, 18 experienced DLC. No significant association was observed between the interval of cholecystitis and DLC using the six-week time factor, as indicated by a p-value of 0.1882 (Table 1).

A history of two or more episodes of cholecystitis was noted in 54 patients: 33 had DLC and 21 had NDLC. The remaining eight patients in the DLC group had a single episode of cholecystitis. Table 1 shows a p-value of 0.0001, indicating a statistically significant association between multiple episodes of cholecystitis and DLC.

Diabetes was present in four patients; DLC occurred in one of these patients (Table 1). A p-value of 0.884 was calculated for diabetes as a risk factor for DLC, indicating no statistically significant association.

Four patients underwent ERCP for choledocholithiasis prior to LC. DLC was encountered in three of these patients. Analysis revealed an unyielding correlation between prior ERCP and DLC, with a p-value of 0.1761 (Table 1).

Three patients had a history of biliary pancreatitis; two of these patients had DLC. All three patients were female and did not have common duct stones on subsequent MRCP. They were treated conservatively without endoscopic intervention and underwent LC after resolution of pancreatitis. No statistically significant association was found between a history of biliary pancreatitis and DLC, with a p-value of 0.7476 (Table 1).

Previous abdominal surgery was present in 33 patients, with most scars being infra-umbilical. DLC was encountered in 19 of these patients. The remaining 22 patients in the DLC group did not have a history of abdominal surgery. A significant association between a history of abdominal surgery and DLC was noted, as indicated by a p-value of 0.0316 (Table 1). Operative difficulty led to the conversion of one case to OC.

GB wall thickness of greater than 3 mm was observed in 18 patients: 12 patients had DLC and 6 had NDLC. Among the remaining 82 patients in both groups with a GB wall thickness of less than 3 mm, 29 had DLC. We found a statistically significant association between GB wall thickness greater than 3 mm and DLC, with a p-value of 0.0292 (Table 2). Difficult GB handling in one patient led to conversion to OC.

**Table 2:** Ultrasonographic features observed. USG: ultrasonography, DLC: difficult laparoscopic cholecystectomy, NDLC: non-difficult laparoscopic cholecystectomy, GB: gallbladder.

USG feature	Number of patients	DLC	NDLC	p-value
GB wall thickness (>3 mm)	18	12	6	0.0292
Presence of multiple stones	70	38	32	0.0001
Stone size (>1 cm)	37	26	11	0.0001
Pericholecystic fluid collection	1	1	0	0.8541
Impacted calculi	6	6	0	0.0093
Liver cirrhosis	1	0	1	0.8541

Seventy patients had multiple GB stones, with operative difficulty observed in 38 patients. DLC was encountered in three patients among 30 patients with a single gallstone (Table 2). Correlating the number of stones with difficult LC was statistically significant, with a p-value of 0.0001 for multiple stones.

The largest gallstone size greater than 1 cm was observed in 37 patients, with operation difficulties encountered in 26 of these patients. The remaining 63 patients had the largest gallstone size of less than 1 cm, and 15 of these patients experienced DLC (Table 2). We found that a stone size greater than 1 cm was a statistically significant indicator of DLC, with a p-value of 0.0001.

Impacted stones were observed in six patients: four were reported on preoperative USG and two were discovered intraoperatively. DLC was observed in all six patients with stones impacted either in the cystic duct or Hartmann’s pouch. The remaining 35 patients in the DLC group had mobile stones. Calculi impaction was found to have a positive association with DLC, with a p-value of 0.0093 (Table 2).

Pericholecystic fluid collection was noted in only one patient, who experienced a difficult LC. Chi-square analysis did not show a significant association between pericholecystic fluid collection and DLC, as the p-value was 0.8541 (Table 2).

There was only one patient with liver cirrhosis, and operative difficulty was not observed. The correlation of liver cirrhosis with DLC did not show a significant association, with a p-value of 0.8541 (Table 2).

Difficulty in port entry was encountered in 14 patients from the DLC group and five patients from the NDLC group (Table 3). The remaining 27 patients in the DLC group had uncomplicated trocar installation. A p-value of 0.0031 was calculated, indicating a statistically significant association between difficult port entry and DLC.

**Table 3:** Intraoperative factors. DLC: difficult laparoscopic cholecystectomy, NDLC: non-difficult laparoscopic cholecystectomy, GB: gallbladder.

Intraoperative findings	Number of patients	DLC	NDLC	p-value
Difficult port entry	19	14	5	0.0031
Bleeding	9	6	3	0.1985
Pericholecystic adhesions	29	22	7	0.0001
Difficult dissection of GB from the liver bed	23	20	3	0.0001
Difficult Calot’s triangle dissection	23	19	4	0.0001
Abnormal anatomy	6	5	1	0.0807
Stone spillage	5	4	1	0.1761
Specimen extraction	27	16	11	0.0425

Bleeding occurred in nine patients: six were from the DLC group and three were from the NDLC group (Table 3). In two patients, bleeding could not be controlled laparoscopically, necessitating conversion to OC. Correlating bleeding as a factor for DLC was not found to be statistically significant, with a p-value of 0.1985.

Anatomical variations were present in six patients, five of whom had DLC (Table 3). The remaining 94 patients had typical anatomy. Common aberrations observed included anomalies in the arterial system (e.g., low-lying cystic artery) and the cystic duct (e.g., low insertion and short cystic duct). A p-value of 0.0807 was observed when correlating abnormal anatomy with DLC, suggesting no statistically significant association.

Pericholecystic adhesions were encountered in 29 patients: 22 were from the DLC group and 7 were from the NDLC group. Statistical analysis of adhesions against DLC demonstrated a significant association, with a p-value of 0.0001 (Table 3).

Difficulty in dissecting the GB from the liver bed was encountered in 23 patients. Twenty patients were in the DLC group, and three were in the NDLC group. The remaining 21 patients in the DLC group had straightforward GB dissection. A p-value of 0.0001 indicated a statistically significant correlation between difficult GB dissection and DLC (Table 3).

In 23 patients, difficulty was experienced in establishing Strasberg’s critical view of safety. Difficult dissection at the hepatocystic/Calot’s triangle was encountered in 19 patients from the DLC group and four patients from the NDLC group. In three cases, dense adhesions at Calot's triangle necessitated conversion to OC. The correlation of DLC with difficult dissection of the hepatocystic triangle was found to be statistically significant, with a p-value of 0.001 (Table 3).

Stone spillage occurred in four patients from the DLC group and one patient from the NDLC group. The correlation of DLC with stone spillage was not found to be statistically significant, with a p-value of 0.1761 (Table 3).

Difficulty in specimen extraction was encountered in 27 cases: 16 were from the DLC group and 11 were from the NDLC group (Table 3). The correlation between difficult specimen extraction and DLC was found to be statistically significant, with a p-value of 0.0425.

## Discussion

A male-to-female ratio of 1:10.1 was noted in our study, suggesting a female preponderance of gallstone disease, consistent with published literature. We found the male gender to be a significant factor correlating with DLC, with a p-value of 0.0459, aligning with findings by Saleem et al. [10]. In some series, male sex was not identified as an independent or isolated predictor of difficult LC [11,12]. However, Akcakaya et al. and Kologlu et al. noted that LC was more challenging in men [13,14].

A BMI greater than 30 kg/m² was found to have a significant association with DLC, with a p-value of 0.0130, consistent with studies by Rosen et al. and Nuzzo et al. [15,16]. Conversely, Afaneh et al. did not find conversion rates or surgical morbidity of LC to be related to obesity [17].

In our study, the correlation between the interval of cholecystitis attack and DLC was not significant when using a six-week time factor (p-value of 0.1882). Similar findings were reported by Bhandari et al. and Nidoni et al. [18,19]. We acknowledge that, in actual practice, other causes of abdominal pain misinterpreted as cholecystitis may act as confounders in the relationship between "episodes of cholecystitis" and DLC.

We observed a significant association between a history of two or more attacks of acute cholecystitis (AC) and DLC, with a p-value of 0.0001. Stanisic et al. cited more than five episodes of pain attacks as a predictor of DLC [20]. Our findings are similar to those of Kologlu et al., Nidoni et al., and Santharaj et al., who concluded that patients with more than two attacks had higher rates of difficulty and conversion compared to those with fewer than two attacks of cholecystitis [14,19,21].

Diabetes as a risk factor for DLC was not evident in our study (p-value of 0.8845), consistent with observations by Zayd et al., who reported that diabetes is not an independent factor for DLC, and diabetic patients did not have increased conversion to OC [22]. Some authors, however, have described an increased conversion to laparotomy in diabetics undergoing LC [23,24]. The limited number of diabetic patients in our study may partially explain our findings.

Although a perceptible difference in technical difficulty was noted, a statistically significant association between post-ERCP status and DLC was not detected (p-value of 0.1761). Prajapati et al. found LC to be more challenging when performed two or more weeks after ERCP [25]. Other authors reported that performing LC one to three days after ERCP can reduce intraoperative difficulty [26-28]. In our study, operations were conducted within one week following ERCP. However, the small number of post-ERCP cases may limit the robustness of our findings.

We did not find biliary pancreatitis to be a risk factor for DLC, with a p-value of 0.7476. Whether our results reflect a milder degree of pancreatitis in our patients or are due to the small number of cases in our study remains uncertain. Bansal et al. reported increased operative difficulty in LC associated with biliary pancreatitis [29]. Da Costa et al. advised anticipating a difficult cholecystectomy after mild gallstone pancreatitis in some cases, including male patients [30]. However, Maitra et al. argued that "cholecystectomy should not be difficult in pancreatitis patients as the primary pathology in gallstone pancreatitis is in the pancreas and not in the gallbladder" [31].

A statistically significant association was observed between previous abdominal operations and DLC, with a p-value of 0.0316, consistent with findings by Nakeeb et al. and Griniatsos, who cited previous abdominal operations as a predictive factor for conversion to OC [32,33]. Extensive adhesions in one case with a ventriculoperitoneal shunt led to conversion to OC. We recognize that the pattern of adhesions formed from previous surgery was not easy to predict.

Only one patient with liver cirrhosis was present in our study, and this patient underwent a NDLC, with a p-value of 0.8541. A large retrospective cohort study by Nakeeb et al. cited cirrhotic liver status as being associated with primary OC or conversion to OC [32]. Griniatsos, in his review article, stated that a higher conversion rate should be expected [33]. Due to the limited number of patients with liver cirrhosis in our study, we cannot convincingly argue that liver cirrhosis is not associated with DLC.

A p-value of 0.0292 was obtained correlating GB wall thickness greater than 3 mm with DLC. A thick GB wall led to difficulty grasping and retracting the GB, preventing optimal visualization of Calot’s area anatomy and making dissection technically challenging, with an increased risk of injury to adjacent structures. One case required conversion to OC. Our findings align with observations by Khan et al., who considered a thick GB wall as greater than 2 mm and stated that GB wall thickening is a strong predictor for outcomes following LC [34]. Some authors have defined a thick GB wall as greater than 4 mm on USG [18,20,21]. Majeski et al. stated that the presence of gallstones and a GB wall thickness of ≥3 mm portends DLC [35].

A positive correlation between multiple stones and DLC was noted, with a p-value of 0.0001, consistent with findings by Hassan et al. [36]. Jansen et al. did not find the number of GB stones to correlate with DLC [37]. Multiple stones tend to tightly pack and distend the GB, reducing its grasping and retraction capability, thereby prolonging operation time regardless of whether spillage occurred.

Only one patient had pericholecystic fluid. We did not find a statistically significant correlation between pericholecystic fluid and DLC, with a p-value of 0.8541. However, the ability to draw a meaningful conclusion is limited by the incidence of only a single case. Gupta et al. did not find pericholecystic fluid to be a factor for DLC [3]. Chindarkar et al., however, mentioned pericholecystic fluid as a factor associated with DLC [38].

A positive correlation between gallstone size greater than 1 cm and DLC was found, with a p-value of 0.0001, akin to findings by previous authors [29,36,38]. A stone size greater than 1 cm interfered with GB grasping, particularly when the GB was contracted. We observed that large stones posed additional challenges during specimen extraction.

We found a strong association between impacted gallstones and DLC, with a p-value of 0.0093, consistent with results from previous studies [39,40]. Stone impaction at the GB neck poses technical challenges, including insufficient grip on the GB, poor exposure of Calot’s area, and inadequate delineation of hepatocystic anatomy.

A statistically significant association between difficult port entry and DLC was observed, with a p-value of 0.0031, echoing observations by Vivek et al., who identified difficult port entry in patients with a BMI >30 kg/m² and those with previous abdominal surgery [9]. Trocar site bleeding and trocar-induced omental injury were managed using monopolar energy or suturing. Malik et al., in their retrospective analysis of 1,046 cases, reported an incidence of access-related complications of 3.77%, including troublesome port-site bleeding requiring re-do laparoscopy or conversion [41].

No statistical correlation was found between abnormal anatomy and DLC, with a p-value of 0.0807. No patients sustained vascular or ductal injury. Continuous assessment of the surgical field and the revision of ambiguous anatomy prolonged operating times. The observed anomalies included low-lying cystic arteries, low insertion of the cystic duct, and short cystic ducts. Ding et al. reported frequent anatomical variation in and around Calot’s triangle in 20%-50% of patients [42]. Singh et al. recommend identifying the cystic lymph node as a landmark for defining the cystic duct and cystic artery [43].

A positive correlation was noted between difficult GB dissection and DLC, with a p-value of 0.0001, aligning with observations by Chhaparia et al. [44]. In two cases, part of the GB's posterior wall was left in situ, with the mucosa fulgurated. Gode et al. advocated Type I laparoscopic subtotal cholecystectomy (LSTC) as a safe procedure for difficult GB bed dissection [45]. Thickened, partially buried, and contracted GBs contributed to the difficulty in mobilizing the GB from the liver bed.

Bleeding occurred from the cystic artery, port sites, omentum, and liver bed, necessitating conversion to OC in two patients. Murod et al., in their systematic review, quoted bile duct injury and uncontrollable bleeding as the most common surgeon-related intraoperative causes of conversion in LC [46]. We did not find a significant association between bleeding and DLC, with a p-value of 0.1985. The proficiency of the surgeons and the limited number of cases with bleeding complications may explain this result.

Only dense adhesions between the GB and surrounding organs, including omental adhesions that required separation by scissors or electrocautery, were considered. A positive correlation with DLC was observed, with a p-value of 0.0001. Griniatsos mentioned dense pericholecystic adhesions as one of the most recognizable causes of conversion to laparotomy [33]. Gupta et al., in their prospective randomized study, described denser adhesions and longer surgical times in patients undergoing elective LC compared to those who had LC within five days after acute cholecystitis [47].

We found a strong association between difficult dissection of Calot’s triangle and DLC, with a p-value of 0.0001. The fundus-first technique was employed electively, and three cases were converted to OC. A fibrotic response in the fibro-fatty tissue (referred to as "Frozen Calot’s Δ") made the dissection of structures challenging due to the obliteration of cleavage planes. Previous authors have noted that dense adhesions at Calot’s triangle predispose patients to DLC and increase the likelihood of conversion to OC [3,18]. Hussain recommends using the body-first or dome-down technique to identify the GB neck and cystic duct, thereby reducing the risk of iatrogenic injury [6].

Gallstone spillage did not correlate with DLC, with a p-value of 0.1761. In a prospective study of 210 patients, Gupta et al. cited spillage of bile or stones as a marker for DLC, though not for conversion to OC [3]. Some authors have used bile or stone spillage as a postoperative parameter to categorize LC as easy or difficult [48]. We applied clips to approximate the edges of the GB wall perforation and successfully controlled bile and stone spillage.

A positive correlation was found between difficult GB extraction and operative difficulty, with a p-value of 0.0425, consistent with the observations of Vivek et al., who identified difficult GB extraction as a factor contributing to operative challenges [9]. Cases involving large gallstones, thickened GBs, and obesity required an extension of the port site incision to facilitate GB extraction. Bordelon et al. stated that protracted attempts at stone crushing or the use of expensive stone fragmentation devices are unnecessary for extracting a difficult GB during LC; instead, extending the fascial incision to remove the impacted GB was time-efficient and did not increase postoperative pain [49].

Operative outcome

In our study, the rate of conversion to OC was 7%. Difficult Calot’s triangle dissection accounted for three cases, bleeding was the reason in two cases, and previous abdominal surgery and a thick GB wall were each responsible for two cases. Of the total seven patients, three were male. Two patients underwent LSTC.

Limitations of the study

The limitations include a small sample size, being a single-center study, the influence of surgeon experience, the absence of multivariable analysis for independent risk factors, the subjective nature of the definition of DLC, and the use of operating time as the determinant for qualifying a laparoscopic cholecystectomy as difficult.

## Conclusions

An overlap of multiple factors and their complex interplay led to the non-progression of LC, necessitating the adoption of bail-out procedures or conversion to open surgery. We observed that intraoperative factors were the most critical reasons influencing decisive actions concerning the course and outcome of LC. Swift conversion to open surgery proved to be the best course of action in cases of critical bleeding, as well as to manage difficult dissection caused by dense adhesions in Calot’s triangle or the pericholecystic environment. LSTC emerged as a safe and feasible alternative to OC for challenging dissection of the GB from the liver bed. Conversion to OC and performing LSTC have enabled us to provide safe and effective treatment for GB lithiasis without complications.

Preoperative factors associated with DLC included male gender, BMI greater than 30 kg/m², a higher number of previous attacks, prior abdominal surgery, calculi number, calculi size, calculi impaction, and a thick GB. Conversion to OC, however, was primarily related to prior abdominal surgery and a thick GB.

Awareness of these features and their potential predisposition to DLC will equip the surgical team with sufficient information to anticipate difficulties, offer preoperative counseling regarding the likelihood of conversion to laparotomy, and develop a plan of action that ensures the allocation of experienced surgeons and the best available tools to make LC as safe as possible.

An aptitude for crisis recognition, coupled with a low threshold for conversion to open surgery, will enable surgeons to make prudent decisions for performing laparoscopic cholecystectomy safely, exemplifying the guiding principle of “primum non nocere,” not viewing conversion to open surgery as a failure, but rather applauding it for prioritizing patient safety.
